# Role of PARP1-mediated autophagy in EGFR-TKI resistance in non-small cell lung cancer

**DOI:** 10.1038/s41598-020-77908-z

**Published:** 2020-12-01

**Authors:** Zhimin Zhang, Xiaojuan Lian, Wei Xie, Jin Quan, Maojun Liao, Yan Wu, Zhen-Zhou Yang, Ge Wang

**Affiliations:** 1 Cancer Center, Daping Hospital, Army Medical University, #10 Changjiang Zhilu, Daping, Yuzhong District, Chongqing, 400042 China; 2 Department of Oncology, Jiangjin Strict Central Hospital, Chongqing, 402260 China; 3 Department of Oncology, Second Hospital Affiliated to Chongqing Medical University, #76 Linjiang Road, Chongqing, 40010 China

**Keywords:** Cancer, Cancer

## Abstract

Resistance to epidermal growth factor receptor-tyrosine kinase inhibitors (EGFR-TKIs) has become the main clinical challenge of advanced lung cancer. This research aimed to explore the role of PARP1-mediated autophagy in the progression of TKI therapy. PARP1-mediated autophagy was evaluated in vitro by CCK-8 assay, clonogenic assay, immunofluorescence, and western blot in the HCC-827, H1975, and H1299 cells treated with icotinib (Ico), rapamycin, and AZD2281 (olaparib) alone or in combination. Our results and GEO dataset analysis confirmed that PARP1 is expressed at lower levels in TKI-sensitive cells than in TKI-resistant cells. Low PARP1 expression and high p62 expression were associated with good outcomes among patients with NSCLC after TKI therapy. AZD2281 and a lysosomal inhibitor reversed resistance to Ico by decreasing PARP1 and LC3 in cells, but an mTOR inhibitor did not decrease Ico resistance. The combination of AZD2281 and Ico exerted a markedly enhanced antitumor effect by reducing PARP1 expression and autophagy in vivo. Knockdown of PARP1 expression reversed the resistance to TKI by the mTOR/Akt/autophagy pathway in HCC-827IR, H1975, and H1299 cells. PARP1-mediated autophagy is a key pathway for TKI resistance in NSCLC cells that participates in the resistance to TKIs. Olaparib may serve as a novel method to overcome the resistance to TKIs.

## Introduction

Epidermal growth factor receptor-tyrosine kinase inhibitors (EGFR-TKIs) are increasingly available and are used in the treatment of patients with non-small cell lung carcinoma (NSCLC) harboring EGFR-activating mutations^1^, but acquired resistance to TKIs is a growing problem that leads to a poor prognosis^2^. Overcoming resistance to TKIs is therefore an important clinical issue. TKIs combined with radiotherapy or other conventional cancer therapies do not improve prognosis^3^. Different strategies have been suggested to overcome this resistance, such as combining TKIs with thermal therapy^4^, anti-angiogenic agents^5,6^, or c-Met inhibitors^7^. These strategies seem to improve prognosis, but the results are far from perfect. Thus, novel targets are still required to overcome resistance.

Autophagy is a process in which damaged organelles, protein aggregates, and bacteria are digested in autophagic vesicles^8^. The aim of autophagy is to maintain a homeostatic state by degrading organelles and proteins^8^. Autophagy is a protective, cellular response to stress that is upregulated following hypoxia, starvation, and drug exposure^9^. Autophagy is upregulated in cancer cells because of their high metabolic state to maintain energy balance and prevent apoptosis^10^. Autophagy is upregulated in the presence of a number of cancer drugs, including TKIs^11–15^, and plays a critical role in the resistance to TKIs through Atg5 but not Beclin 1^16,17^. Treatment with autophagy inhibitors combination with TKIs may be an effective approach when basal autophagy is upregulated in cancer cells^18–20^. Inhibiting autophagy overcomes TKI resistance through the modulation of endoplasmic reticulum stress and apoptosis^9^.

We detected the levels of different molecules downstream of EGFR and found that the expression of mTOR/p-Akt was significantly different in cancerous and paracancerous tissues, but the expression of PI3K was not different. Poly(ADP)-ribose polymerase-1 (PARP1) repairs single-strand breaks in DNA and is important in autophagy regulation^21^. Reactive oxygen species (ROS) induce PARP1 activity and autophagy, and PARP1 knockout cells display low levels of autophagy, suggesting a prosurvival role for autophagy and PARP1 activity^22^. PARP1 is an important, positive regulator of autophagy induced by DNA damage, ROS, and starvation^22,23^. PARP1 activation and ATP depletion mediate cell death and autophagy via AMPK activation and mTOR suppression^24^. Furthermore, PARP1 inhibitors have antitumor activity in some types of cancer^25^. The poly-ubiquitin receptor p62 is also involved in the regulation of PARP1-mediated autophagy^26^. Therefore, treatment with a combination of TKIs and PARP1 inhibitors could be a potential approach to overcome TKI resistance and improve prognosis, but the exact involvement of PARP1 in the autophagy of NSCLC cells is still poorly understood. Therefore, the aim of the present study was to explore the role of the PARP1-mediated autophagy pathway in TKI resistance in NSCLC.

## Results

### TKI treatment increases the expression of PARP1 and LC3 II in TKI-resistant HCC-827IR and H1299 cells

**Figure 1:**
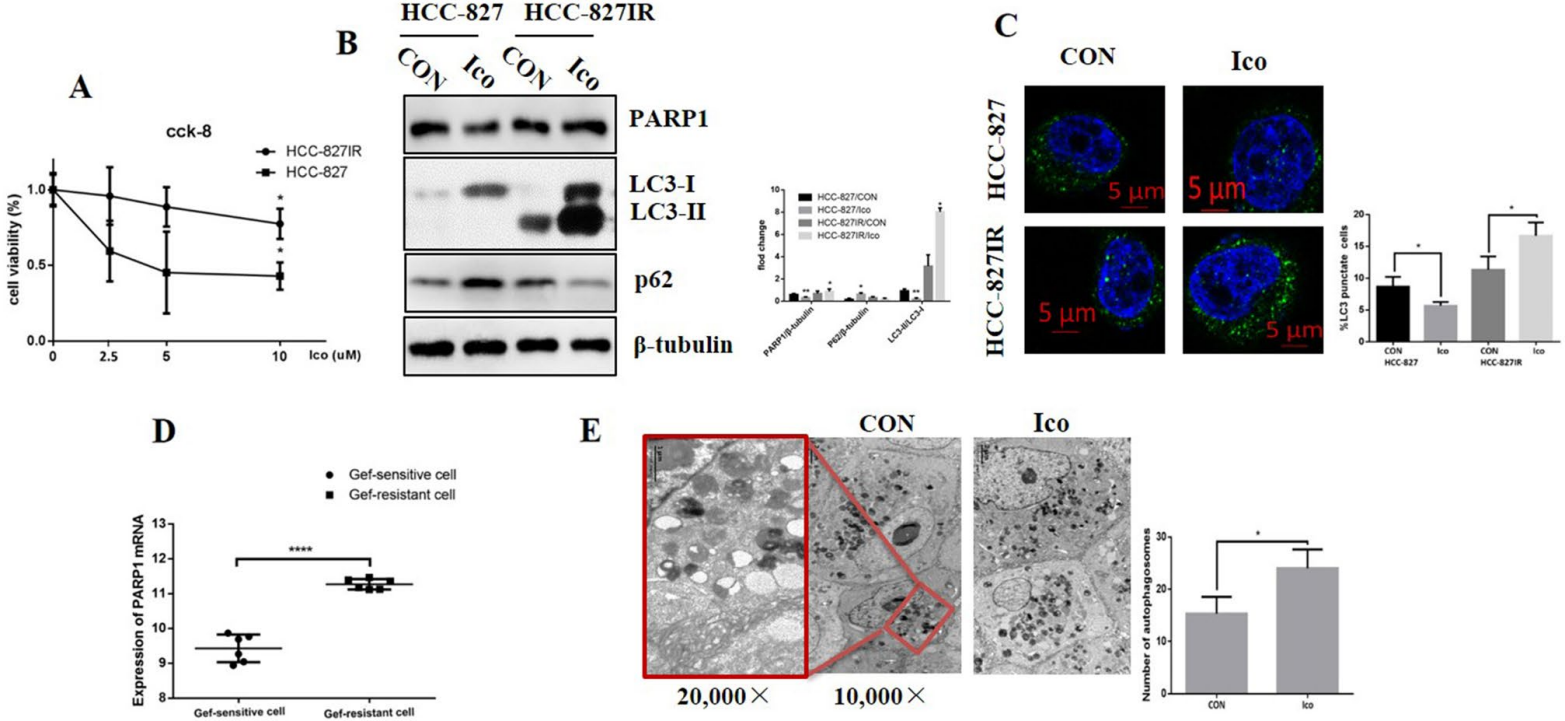
Expression of PARP1 and autophagy in TKI-resistant cells. (**A**) HCC-827 and HCC-827IR cells were treated with 0, 2.5, 5, or 10 µM Ico for 48 h, CCK-8 assay measured the cell viability. (**B**) HCC-827 and HCC-827IR cells were treated with 5 µM Ico for 48 h, western blot tested the expression of PARP1, LC3 and P62. The data represent means ± SD of three independent experiments. *P < 0.05 versus untreated **P < 0.001 versus untreated (Because of the films before imaging were cut, we couldn’t further provided fuller-length original blots). (**C**) HCC-827 cells and HCC-827IR cells were transfected with GFP-LC3; 24 h following transfection, cells were rapa treatment. With or without Ico treatment. Treatment with 100 nM rapamycin for 4 h was used as positive control of autophagosome accumulation. From 50 cells analyzed for each condition, the pictures in the right panel show representative images with the subcellular distribution of the autophagic vesicle marker LC3. *P < 0.001 comparing between HCC-827 cells and HCC-827IR cells. (**D**) Data analysis from GEO dataset of PARP1 mRNA in TKI-sensitivity cells and TKI-resistance cells. One-way ANOVA analysis comparing the mRNA level of PARP1 in 2 TKI-sensitive cell lines and 2 TKI-resistant cell lines, with a total of 4 samples in triplicate analyzed. (E) Electron microscope assay tested the autophagosome level after Ico treatment in HCC-827IR cells. The data represent means ± SD of three independent experiments. *P < 0.05 versus untreated **P < 0.001 versus untreated.

TKI resistance is common in NSCLC, identifying the differences between TKI-sensitive and TKI-resistant cells is needed to better understand its mechanisms. HCC-827 was shown to be sensitive to icotinib (Ico) and HCC-827IR was shown to be resistant to Ico using the CCK-8 assay (Fig. 1A). Ico treatment revealed that PARP1 and LC3 II/ LC3 I ratio was decreased in HCC-827 cells but increased in HCC-827IR cells compared to that observed without treatment. The autophagy substrate, p62 expression, was negative correlated with LC3 II level (Fig. 1B). To further confirm the Fig. 1B results and show the differences of autophagy level between TKI-sensitive and TKI-resistant cells, we repeated the above process and transiently transfected HCC-827 cells and HCC-827IR cells with GFP-LC3 and treated these cells with or without Ico. The percentage of cells with punctate pattern of GFP-LC3 was counted by fluorescence microscopy. The punctate pattern (an accumulation of autophagosomes) in HCC-827IR cells were significantly higher than HCC-827 cells, and after treatment with Ico there was more punctate pattern (Fig. 1C). To identify the differences of PAPR1 expression between TKI-sensitive and TKI-resistant cells, data analysis from GEO dataset of PARP1 mRNA in TKI-sensitivity cells and TKI-resistance cells. One-way ANOVA analysis comparing the mRNA level of PARP1 in 2 TKI-sensitive cell lines and 2 TKI-resistant cell lines, with a total of 4 samples in triplicate analyzed. We found the mRNA level of PARP1 was significantly higher in TKI (Gef, Gefitinib)-resistant cell lines than that in TKI (Gef)-sensitive cell lines after TKI treatment, as confirmed by GEO dataset analysis (Fig. 1D). And, we also got similar results after Ico treatment in HCC-827 cells (Supplementary Fig. 1B). Finally, to directly observe the differences of autophagy level, from 50 cells analyzed for each condition, electron microscope assay tested the autophagosome level after Ico treatment in HCC-827IR cells. Electron microscope confirmed that HCC-827IR cells had higher level of autophagy by Ico treatment than control group (Fig. 1E). These results suggest that the trend toward PARP1 expression and autophagy was clearly different in the HCC-827 and HCC-827IR cells after TKI treatment, which could be attributed to Ico resistance in HCC-827IR.

### PARP1 and p62 expression were associated with PFS and OS in TKI-treated NSCLC patients

**Figure 2:**
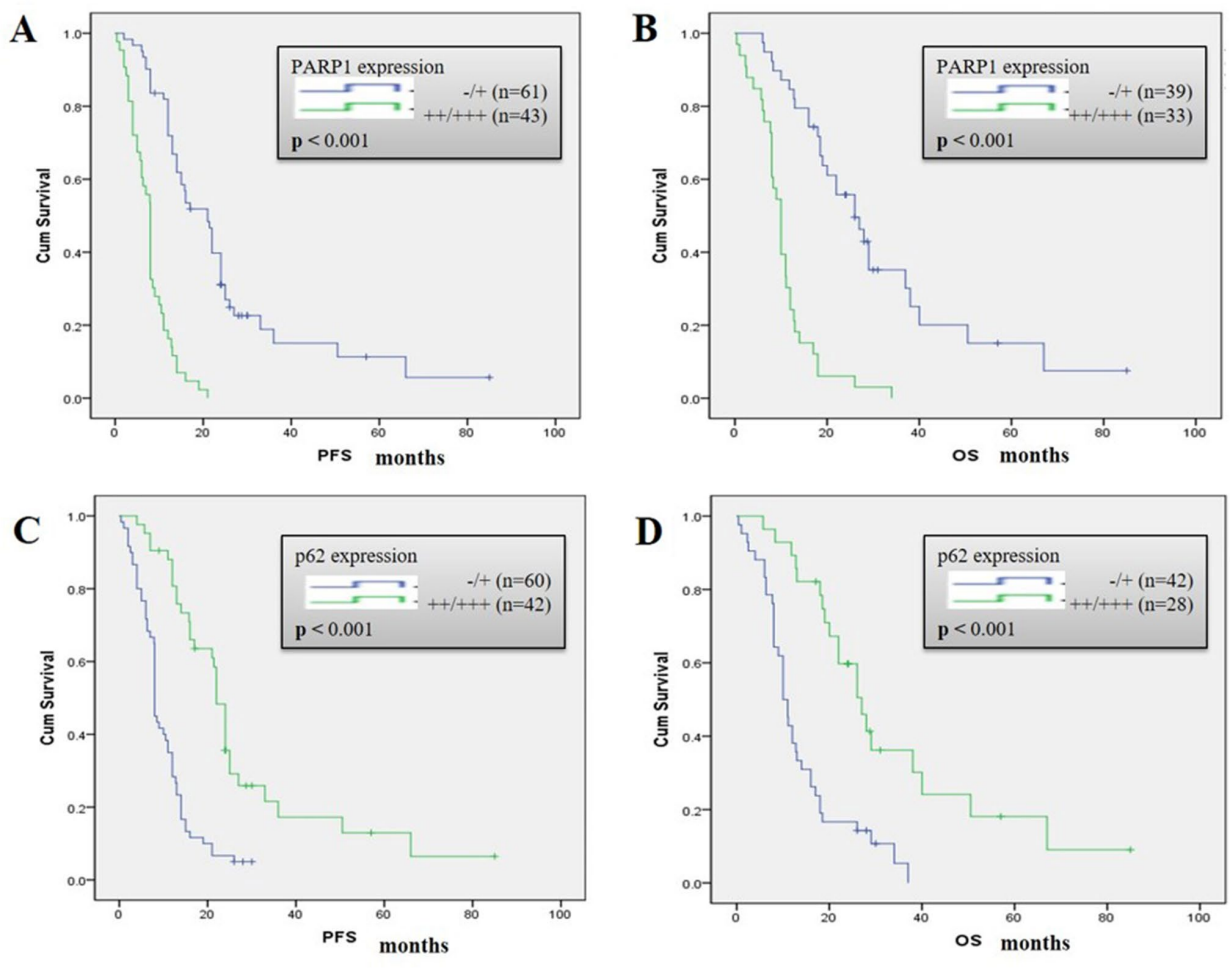
Role of the PARP1-autophagy signaling pathway in progression-free survival (PFS) and overall survival (OS) after TKI therapy in human non-small lung cancer tissue. (**A**,**C**) Kaplan–Meier analysis comparing the PFS and (**B**,**D**) OS of patients exhibiting high and low expression of PARP1 (**A**,**B**) and p62 (**C**,**D**). *P* values were obtained by the log-rank test.

We evaluated the expression of PARP1 and p62 proteins in 104 patients with NSCLC with an EGFR mutation, and all patients received TKI treatment. PARP1 was distributed in the cytoplasm and nucleus, while p62 was only localized in the cytoplasm. Kaplan‐Meier survival curves show, compared with PFS and OS associated with the low expression of PARP1, high expression of PARP1 was associated with shorter PFS (*P* < 0.005) and OS (*P* < 0.005) (Fig. 2A,B). Compared with PFS and OS of the low p62 expression group, the high expression of p62 was associated with longer PFS (*P* < 0.005) and OS (*P* < 0.005) (Fig. 2C,D). These data suggest that the low expression of PARP1 could be associated with better outcomes for NSCLC patients treated with TKIs, p62 expression was negatively correlated with OS and PFS in patients. These results implied that the PARP1/autophagy may play an important role in the survival outcome of EGFR mutations in patients with NSCLC.

### Role of PARP1-mediated autophagy in Ico resistance

**Figure 3:**
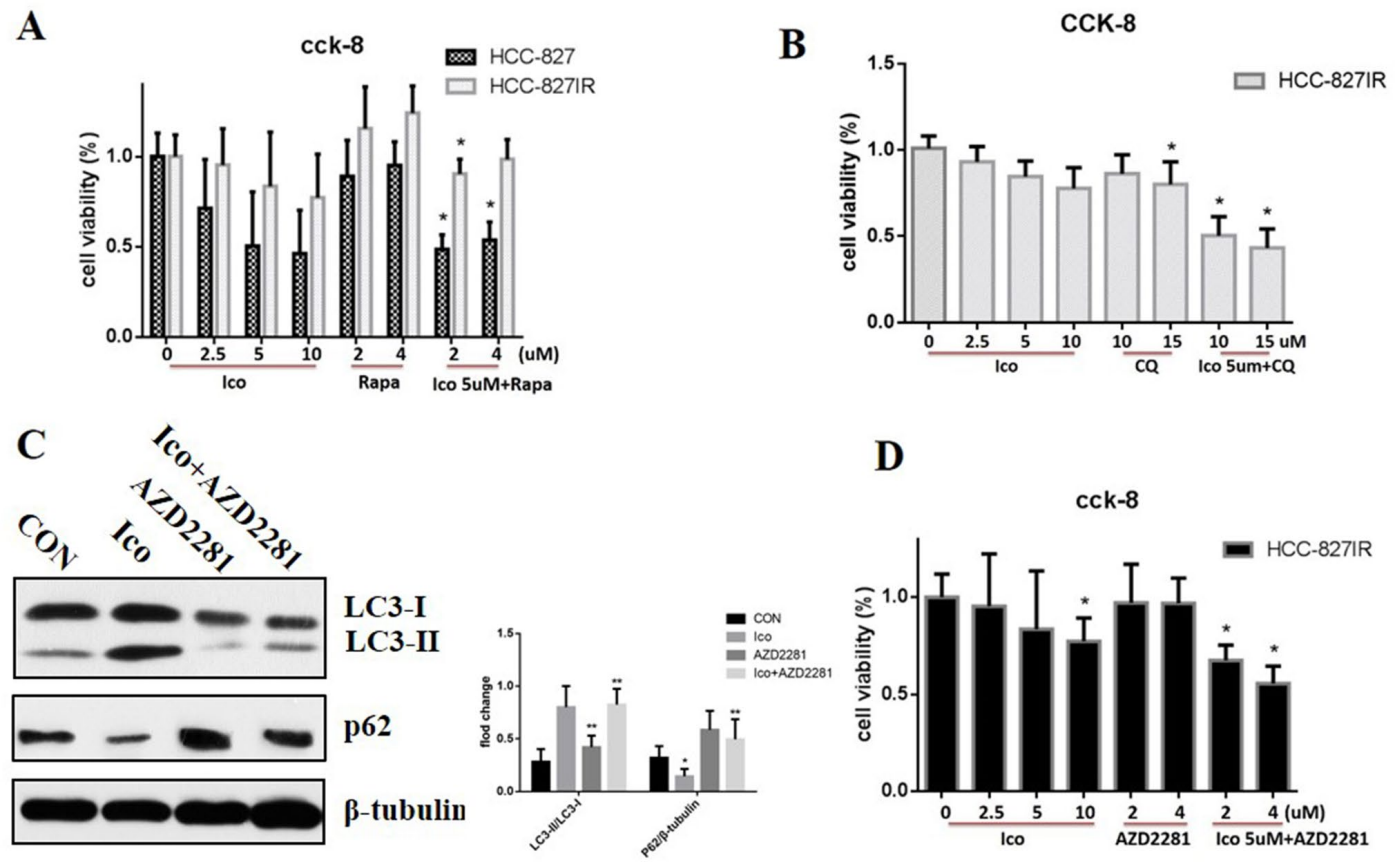
The inhibition of PARP1/autophagy reversed Ico resistance in HCC-827IR cells. (**A**) HCC-827 and HCC-827IR cells were treated with 0, 2.5, 5, or 10 µM Ico; 2 or 4 µM rapamycin; or a combination of both for 48 h. Cell viability was determined by the CCK-8 assay. The columns represent means ± SD of three independent experiments. (**B**) HCC-827IR cells were treated with 0, 2.5, 5, or 10 µM Ico; 10 or 15 µM CQ; or a combination of both for 48 h. Cell viability was determined by the CCK-8 assay. The columns represent means ± SD of three independent experiments. (**C**) HCC-827IR cells were treated with blank, 5 µM Ico, 4 µM AZD2281, or a combination of both for 24 h. western blot measured protein expression. The data represent means ± SD of three independent experiments, *P < 0.05 versus untreated **P < 0.001 versus untreated (because of the films before imaging were cut, we couldn’t further provided fuller-length original blots). (**D**) HCC-827IR cells were treated with 0, 2.5, 5, or 10 µM Ico; 2 or 4 µM AZD2281; or a combination of both for 48 h. Cell viability was determined by the CCK-8 assay. The columns represent means ± SD of three independent experiments.*P < 0.05 versus untreated. (*P* < 0.05).

Rapamycin (Rapa) and hydroxychloroquine (CQ) were used to investigate the role of PARP1-mediated autophagy in Ico resistance. The CCK-8 assay showed that Rap, which promotes autophagy, did not inhibit cell proliferation after Ico treatment in HCC-827 and HCC-827IR cells (Fig. 3A). In contrast, CQ, which inhibits autophagy, significantly reduced cell proliferation in HCC-827IR cells after Ico treatment (Fig. 3B). And 3-MA (a specific autophagy inhibitors) also clearly inhibited cell proliferation in HCC-827IR cells (Supplementary figure A). These results suggest that autophagy plays an important role in Ico resistance in HCC-827IR cells.

AZD2281 (olaparib) was used to inhibit PARP1. Ico treatment increased the ratio of LC3 II/LC3 I when used alone, while the combination of AZD2281 with Ico decreased the ratio of LC3 II/LC3 I compared to that observed in control cells (Fig. 3C). In addition, the combination of AZD2281 and Ico had a synergetic effect on cell proliferation by increased LC3 II accumulation in HCC-827IR cells (Fig. 3D), compared with that observed in the Ico or control groups (Fig. 1D). Taken together, both CQ and AZD2281 significantly decreased the resistance of HCC-827IR cells to Ico, indicating that PARP1-mediated autophagy is an important mechanism required for Ico resistance.

### PARP1-regulated autophagy is required for TKI resistance in primary TKI-resistant cell lines

**Figure 4:**
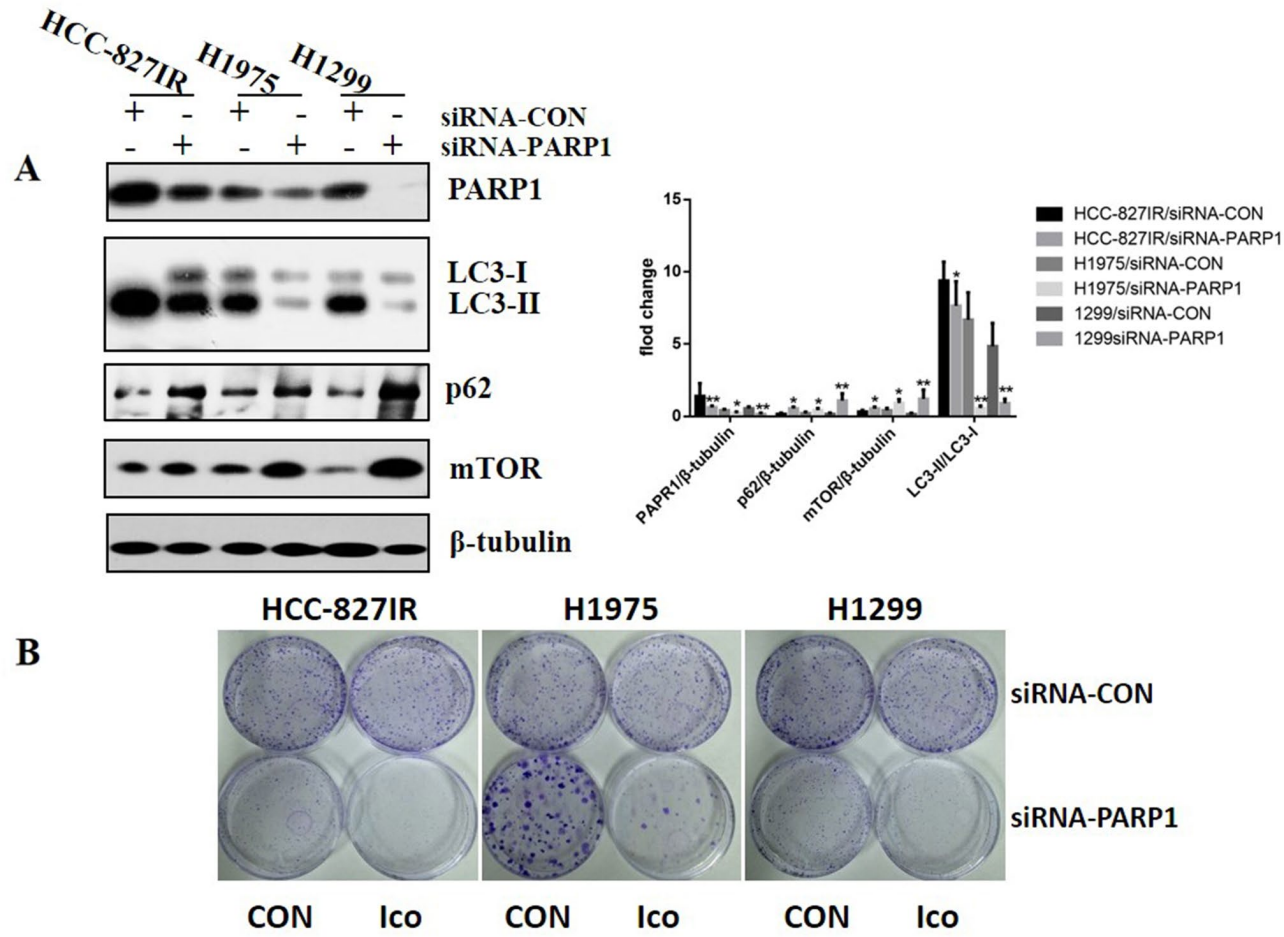
Effects of PARP1 on cell autophagy in HCC-827IR, H1975, and H1299 cells. (**A**) Knockdown of PARP1 by siRNA-PARP1 in HCC-827IR, H1975, and H1299 cells. Western blot measured protein expression. The data represent means ± SD of three independent experiments, *P < 0.05 versus untreated **P < 0.001 versus untreated, (Because of the films before imaging were cut, we couldn’t further provided fuller-length original blots). (**B**) Colony proliferation assay determined cell proliferation. PARP1 in HCC-827IR, H1975, and H1299 cells was knocked down by siRNA. Cell growth was determined by crystal violet staining. The data represent means ± SD of three independent experiments. (*P* < 0.05).

The knockdown of PARP1 increased the ratio of LC3 II/LC3 I and mTOR expression in HCC-827IR, H1299, and H1975 (EGFR p. T790M and EGFR p. L858R) cells compared to that in control cells (Fig. 4A). In addition, the knockdown of PARP1 significantly decreased the proliferation induced by Ico in HCC-827IR, H1299, and H1975 cells compared to that in cells expressing PARP1 (Fig. 4B). These results strongly suggest that PARP1 is essential for autophagy in NSCLC and that decreasing PARP1 expression inhibits autophagy and resistance to TKI.

### AZD2281 reverses Ico resistance in vivo

**Figure 5:**
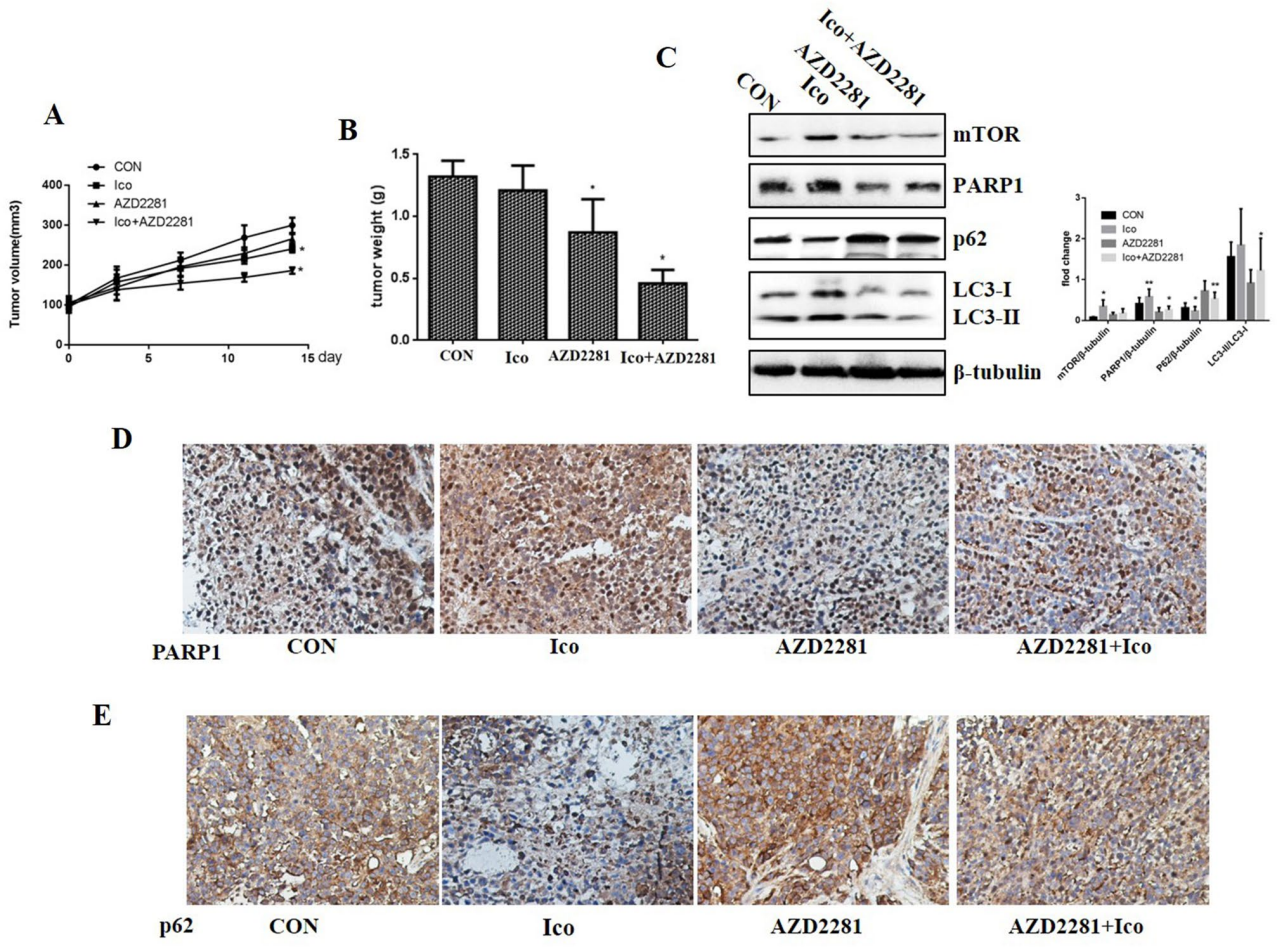
AZD2281 reverses resistance to Ico in vivo. Nude mouse HCC-827IR xenograft models were divided into four groups (10 mice/group) and treated for 14 days with 50 mg/kg Ico once daily and/or 30 mg/kg AZD2281 once daily by intraperitoneal injection. Tumors were resected on day 14. Tumor volumes were measured using a caliper (**A**). Tumor weights were measured on day 14 (**B**). Tumor tissues isolated from xenografts after treatment with Ico, AZD2281, or Ico + AZD2281 were subjected to western blot analysis (**C**) (Because of the films before imaging were cut, we couldn’t further provided fuller-length original blots). The data represent means ± SD of three independent experiments, *P < 0.05 versus untreated **P < 0.001 versus untreated. Tumor tissues isolated from xenografts after treatment with Ico, AZD2281, or Ico + AZD2281 were subjected to immunohistochemical stain of PARP1 (**D**) and p62 expression (**E**). The data shown represent the mean ± standard error, n = 10. *P < 0.05 versus the vehicle group.

The efficacy of the combination of Ico + AZD2281 against tumor growth was tested in the HCC-827IR xenograft model. The combination of Ico + AZD2281 significantly inhibited the growth of the xenograft, as measured by tumor size and weight (*P* < 0.01, Fig. 5A,B). The PARP1-mediated autophagy signaling pathway was verified in the HCC-827IR xenograft model. Western blot analysis showed that AZD2281 treatment increased p62 expression and increased mTOR expression and the ratio of LC3 II/LC3 I compared to those in the control cells (Fig. 5C). IHC revealed p62 expression in the xenograft model, and the results were consistent with those from the western blot analysis (Fig. 5D,E). The in vivo data further showed that the combination of Ico with AZD2281 reverses the acquired resistance to TKIs.

### The combination of TKIs and PARP1 inhibitor reversed TKI resistance

**Figure 6:**
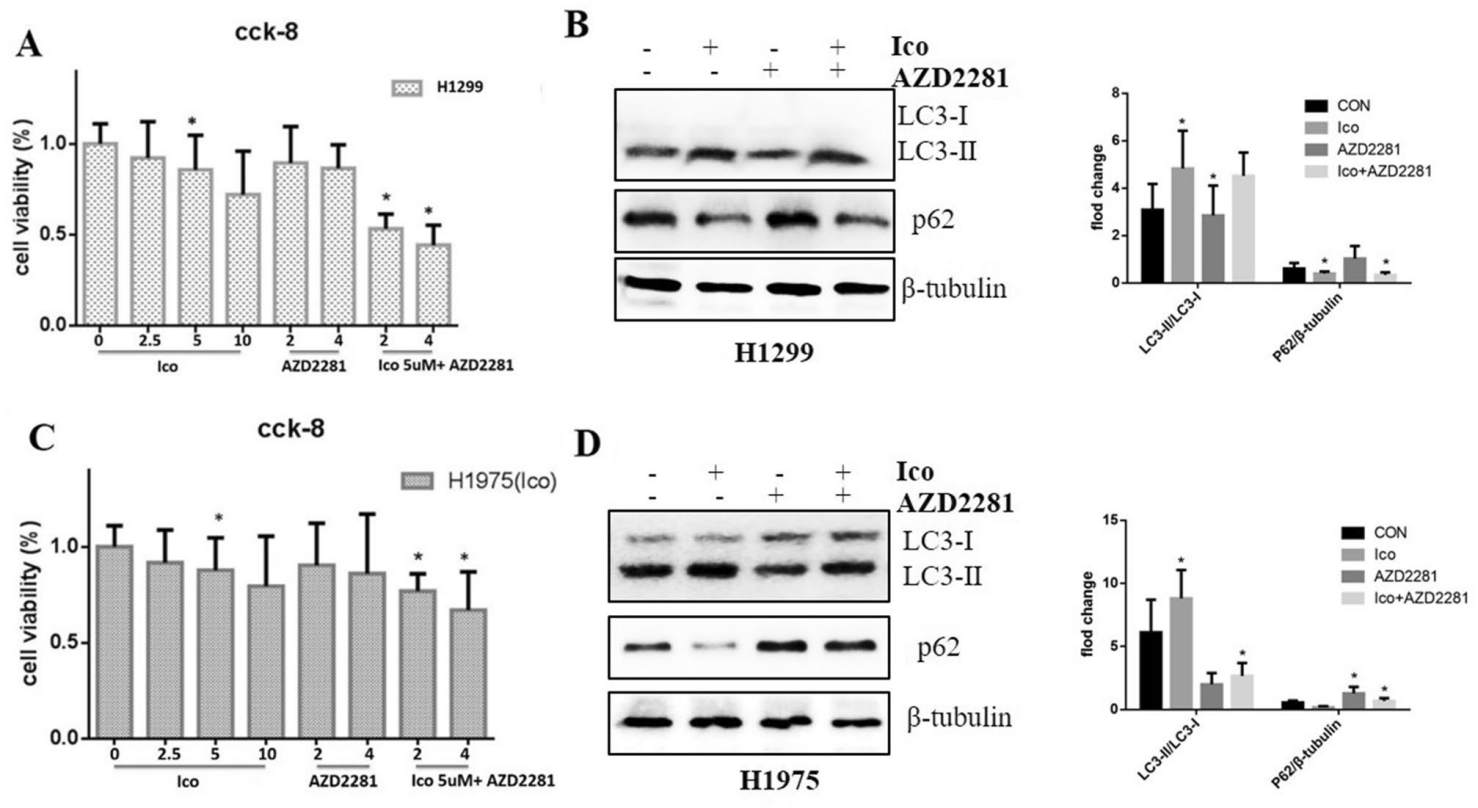
Role of the PARP1 inhibitor Ico in reversing acquired and primary resistance to TKI. (**A**) H1299 cells were treated with 0, 2.5, 5, or 10 µM Ico; 2 or 4 µM AZD2281; or a combination of both for 48 h. Cell viability was determined by the CCK-8 assay. The columns represent means ± SD of three independent experiments. (**B**) H1299 cells were treated with blank, 5 µM Ico, 4 µM AZD2281, or a combination of both. Western blot measured protein expression. The data represent means ± SD of three independent experiments, *P < 0.05 versus untreated (Because of the films before imaging were cut, we couldn’t further provided fuller-length original blots). (**C**) H1975 cells were treated with 0, 2.5, 5, or 10 µM Ico; 2 or 4 µM AZD2281; or a combination of both for 48 h. Cell viability was determined by the CCK-8 assay. The columns represent means ± SD of three independent experiments. (**D**) H1975 cells were treated with blank, 5 µM Ico, 4 µM AZD2281, or a combination of both. Western blot measured protein expression. The data represent means ± SD of three independent experiments. *P < 0.05 versus untreated, (Because of the films before imaging were cut, we couldn’t further provided fuller-length original blots).

To investigate whether AZD2281 affects TKI resistance in NSCLC cells, Ico was used to treat H1975 and H1299 cells. H1299 and H1975 cells, which were not sensitive to Ico, but the addition of AZD2281 increased their sensitivity to Ico (Fig. 6A,C). Immunoblotting showed that AZD2281 decreased the ratio of LC3 II/LC3 I induced by Ico in H1299 and H1975 cells compared to that observed in cells not treated with AZD2281 (Fig. 6B,D). These results suggest that the PARP1-mediated autophagy signaling pathway is probably a critical factor in the resistance to TKIs.

## Discussion

PARP1-mediated autophagy was shown to be a key pathway for TKI resistance in NSCLC cells and to participate in the resistance to TKIs, and olaparib could be a new drug to overcome this resistance. In addition, clinical data showed that longer PFS and OS is clearly associated with lower PARP1 expression and higher p62 expression in patients with NSCLC after TKI therapy compared to their expression levels without TKI therapy. Taken together, these results suggest that the combination of olaparib and TKIs could potentially overcome TKI resistance.

The present study suggests another strategy to reverse resistance to TKIs by the combination of blocking PARP1-induced autophagy and treating with TKIs. Cotreatment with CQ and Gef promoted sensitivity to TKIs by the Akt-mTOR pathway in NSCLC^27^. The inhibition of autophagy led to decreased cetuximab resistance and has been shown to be a novel clinical strategy for TKI therapy^28^. Interestingly, the present study showed that autophagy and PARP1 expression were different in HCC-827 and HCC-827IR cells following TKI treatment. These results were consistent with a study that showed that TKI increases lethal autophagy by altering the relationship between EGFR and Beclin 1 and dissociating Beclin 1/Bcl-2 in HCC-827 and H1975 cells^29^. Nevertheless, Ico treatment induced autophagy and increased PARP1 expression in HCC-827IR cells compared to that in cells without Ico treatment, suggesting that PARP1-mediated autophagy is a prosurvival signaling pathway. In addition, it was proposed that PARP1 enhances autophagy by the ATP- and NAD^+^-mediated AMPK-mTOR pathway^23^. Autophagy mediated by PARP1 activation is a cytoprotective process in response to DNA damage^23^. PARP1 depletion also results in a switch from prosurvival autophagy induced by ROS and starvation to apoptosis^22^. This finding was consistent with the results of the present study, and the clinical data showed a possible association between PARP1 expression and autophagy. A previous study showed that EGFR-activating mutations in NSCLC were associated with PARP1 inhibitor sensitivity^30^, but the detailed mechanisms by which TKIs activate PARP1 still need further investigation. The present study showed that the inhibition of PARP1 using olaparib and siRNA-PARP1 inhibited cell growth and decreased autophagy. PARP1 expression induced by TKIs was different in HCC-827 and HCC-827IR cells, suggesting that the way in which TKIs induce PARP1 activation may involve different signaling pathways.

We propose that PARP1 protein and autophagy levels could be used as biomarkers to predict the emergence of resistance after TKI therapy in NSCLC, as suggested by the xenograft experiment. It was reported that PARP1 expression was negatively associated with breast, ovarian, and pancreatic cancer outcomes^31,32^. The present study suggests a role for PARP1-mediated autophagy in the resistance to TKIs seen in NSCLC. In addition, PARP1 expression was related to p62 expression, suggesting that PARP1 is an upstream molecular regulator of autophagy in human cancers. The detail mechanical between PARP1 and autophagy had been extensively studied^33^. Munoz-Gamez, J. A. etc. found that PARP1 regulated autophagy by the ATP- and NAD + -mediated AMPK-mTOR pathway in human tumor cells^23^. ROS-dependent PARP-1-LKB1-AMPK-mTOR pathway also was proposed in human endothelial cells^34^, Pancreatic ductal adenocarcinoma^35^, Lymphangioleiomyomatosis^36,37^, prostate cancer^38^. In lung cancer the PARP-1-LKB1-AMPK-mTOR pathway also take part in cell death program^39^. Furthermore, olaparib has been shown to be safe and effective in combination therapy^40,41^. The marker of autophagy may be an important indicator for cancer prognosis. Autophagy play a key role in tumorigenesis, p62 is necessary for cell survive and tumorigenicity mediated by Ras^42^. p62 deficiency through mTORC1 inhibition directly supported nutrient availability for cancer cells and promoted tumorigenicity^43^. Our results shown that autophagy substrate, p62 high expression had a longer PFS and OS in patients with NSCLC after TKI therapy. High p62 expression in the tumor area served as a potent indicator for poor outcome of hepatocellular carcinoma patients^44^. SNP rs10898880 in ATG16L2 may be a predictor of RP, LRFS, PFS, and OS in patients with NSCLC after definitive radiotherapy^45^. Clinical trials are necessary to application of PARP1 protein and autophagy marker to the clinic.

Nevertheless, the present study conflicts with previous studies. Indeed, Erguven et al.^29^ showed that EGFR-TKI induced pro-apoptotic autophagy in HCC-827 cells. In addition, the role of autophagy in the induction of apoptosis is the focus of controversy^46^. Based on the present study, we believe that PARP1-mediated autophagy is protective in NSCLC. A variety of uncontrolled environmental factors could shift the delicate balance between autophagy-mediated survival and apoptosis. In addition, in the present study, the combination of TKI with olaparib overcame the resistance of the NSCLC xenografts, as supported by Sui et al.^47^; however, in their study, this phenomenon increased autophagy^47^. Because Sui et al.^47^ did not further explore the molecular mechanisms involved, these differences could be due to the different cell lines used.

Overall, PARP1-mediated autophagy was shown to be a key pathway for TKI resistance in NSCLC cells that participates in the resistance to TKIs. Olaparib may serve as a new drug to overcome NSCLC resistance to TKIs. Our study suggests that PARP1-mediated autophagy is not only a biomarker for TKI treatment outcomes but also a target for reversing TKI resistance. The combined use of a PARP1 inhibitor and an autophagy inhibitor decreased resistance to TKI to some extent, indicating a potential novel therapeutic approach for the treatment of NSCLC.

## Materials and methods

### Materials

Olaparib (PARP1 inhibitor, AZD2281), Rapa and CQ were from Sigma-Aldrich (St Louis, MO, USA). Icotinib (Ico) was provided by Betta Pharmaceutical Co., Ltd. (Hangzhou, China). Both Gef and Ico are first-line choice of EGFR sensitivity mutation and have similar clinical efficacy in NSCLC, however, compared with Gef, Ico has lower IC50 value, shorter half-life and lower adverse drug reactions. Antibodies against p62 and PARP1 were from Santa Cruz Biotechnology (Santa Cruz, CA, USA). Antibodies against mTOR, LC3, LC3II, β-tubulin and β-actin were from Abcam (Cambridge, MA, USA). CCK-8 assay, crystal violet, loading buffer, and RIPA assay were from Beyotime Institute of Biotechnology (Haimen, China).

### Gene expression omnibus (GEO) TKI resistance cell lines datasets

The preprocessed expression data for EGFR TKI resistant EGFR-mutated Cells was download from GEO dataset (https://www.ncbi.nlm.nih.gov/geo/query/acc.cgi/, the number: GSE80344). This dataset (Public on Jul 01, 2016) investigated the gene expression underlying EGFR TKI resistance by using microarrays. Overall design: Total 4 samples in triplicates were analyzed. This dataset was used to identify differentially expressed mRNA between TKI sensitive cell lines and drug resistant cell lines.

### Cell culture

The NHI-HCC-827 (HCC-827, the lung adenocarcinoma cell line has an acquired mutation in the EGFR tyrosine kinase domain (E746–A750 deletion)), NHI-H1975 (H1975, the lung adenocarcinoma cell line has a oncology biomarker EGFR p.T790M and EGFR p.L858R via NGS and ddPCR), and NHI-H1299 (H1299, The cells line have a homozygous partial deletion of the p53 protein, and lack expression of p53 protein.) cell lines were from the American Type Culture Collection (ATCC; Manassas, VA, USA). The Ico-resistant HCC-827 (HCC-827 IR) cell line was kindly provided by the Respiratory Medicine Department of Xinqiao Hospital, Army Medical University. All cells were cultured in containing 10% FBS, 50 mg/mL penicillin/streptomycin and 37 °C humidified incubator with 5% CO_2_.

### Transfection

The siRNA-PARP and siRAN-con plasmids were from Genechem (Shanghai, China). Using the Silencer siRNA construction kit (Qiagen, Venlo, The Netherlands), the cells were incubated with siRNA transfection complexes, and were performed with the RNAiFect reagents (Qiagen, Venlo, The Netherlands). The cells were used for the experiments 48 h after transfection^48^.

### Western blot

Protein extracted using the RIPA buffer. Then determining Protein concentration. Equal amounts of proteins were separated on gels and transferred to membranes. The membranes were blocked for 1 h at RT (room temperature). The membranes were incubated with the primary antibody, then followed by 1 h at RT with the secondary antibodies. Protein bands were visualized with films (Eastman Kodak Co., Rochester, NY, USA). The protein bands were analyzed (Bio-Rad, Hercules, CA, USA).

### Cell counting kit-8 (CCK-8) assay

Cells were seeded in 96-well plates. 24 h after transfection, cell viability was detected according to the manufacturer's instruction. The absorbance was determined (Bio-Rad, Hercules, CA, USA).

### Clonogenic assay

The assay was used to determine the survival and proliferation of the different cell lines after drug treatment^49^. Cells were plated on 60-mm plastic dishes 1 day before drug treatment in order to produce approximately 500 surviving colonies. These cells were treated for 14 days. The cells were stained and counted.

### In vivo experiment

Animal handling were approved by Ethics Committee of Army Medical University. Female BALB/C nude mice (6–8 weeks of age) were obtained from the Daping Hospital of Army Medical University Laboratory Animal. These mice were housed, then injected HCC-827IR cells. Ico was intraperitoneally administered at 50 mg/kg/day, for 3 weeks. AZD2281 was intraperitoneally administered at 30 mg/kg/day for 3 weeks. These mice were measured tumor diameter and tumor size. Mice with tumors reaching about 100 mm^3^ were randomized into four groups (10/group): control (PBS), 50 mg/kg Ico, 30 mg/kg AZD2281, and Ico + AZD2281^47^.

### Electron microscopy

Cell were washed and centrifuged, then cells were stained with 2% uranyl acetate in water in the dark. Sequential ethanol series were used to dehydrate the samples. Sections were made (70 nm) and stained with lead citrate and uranyl citrate. Zeiss microscope performed transmission electron microscopy analysis (Carl Zeiss GmbH, Oberkochen, Germany).

### Immunohistochemistry (IHC)

The slides were baked, blocked and incubated with PARP1 and p62 monoclonal antibody (1:100 and 1:50 dilution), and then with secondary antibody (1:50 dilution). Finally, the slides were incubated with 3,30-diaminobenzidine (DAB). Cytoplasmic PARP1 and p62 staining score was performed by three pathologists^50^. Weak staining and < 50% of stained cells were defined as low expression^51^.

### Patients

The tumor blocks were from 88 patients treated at Daping Hospital of the Army Medical University (China) and 31 patients treated at Wuhan Union Hospital (China) (supplementary table. Clinical characteristics of NSCLC patients). These samples of patients were collected after TKI therapy. According to the World Medical Association Declaration of Helsinki, and that all subjects provided written informed consent, the Ethics Committee of the Daping Hospital, Army Medical University also approved it, and obtaining informed consent from all subjects.

The research was carried out in accordance with the World Medical Association Declaration of Helsinki, and that all subjects provided written informed consent. It was carried out after approval by the Ethics Committee of the Daping Hospital and Research Institute of Surgery (Medical Research Institute (2020) No. 119), Army Medical University and obtaining informed consent from all subjects. The methods in treating tissues were carried out strictly in accordance with institutional policies and approved guidelines of experiment operations. And animal handling were approved by Ethics Committee of Army Medical University.

### Autophagy assay

Cells were transfected with GFP-LC3 vector and jet PEITM according to the manufacturer's protocol. Assays were performed on cells grown on glass coverslips in a six-well plate and treated with HANK buffer or/and rapamycin. The fluorescence image was observed with a Zeiss fluorescence microscope.

### Statistical analysis

Categorical data were presented as frequencies and analyzed using the Fisher exact test. OS and PFS were analyzed using the Kaplan–Meier method and the log-rank test. SPSS 20.0.0 (IBM, Armonk, NY, USA) was used for statistical analysis. Two-sided *P* values < 0.05 were considered statistically significant.

### Ethics declaration

The research was carried out in accordance with the World Medical Association Declaration of Helsinki, and that all subjects provided written informed consent. It was carried out after approval by the Ethics Committee of the Daping Hospital and Research Institute of Surgery (Medical Research Institute (2020) No. 119), Army Medical University and obtaining informed consent from all subjects. The methods in treating tissues were carried out strictly in accordance with institutional policies and approved guidelines of experiment operations. And animal handling were approved by Ethics Committee of Army Medical University.

## Supplementary information

Supplementary Information 1.

Supplementary Information 2.

## Abbreviations

EGFR-TKIs: Epidermal growth factor receptor-tyrosine kinase inhibitors
olaparib: AZD2281
Ico: Icotinib
NSCLC: Non-small cell lung carcinoma
PARP1: Poly ADP-ribose polymerase-1
ROS: Reactive oxygen species
CQ: Hydroxychloroquine
Rapa: Rapamycin
GEO: Gene expression omnibus
HCC-827: NHI-HCC-827
H1975: NHI-H1975
H1299: NHI-H1299
ATCC: American type culture collection
HCC-827 IR: The Ico-resistant HCC-827
RT: Room temperature
CCK-8: Cell counting kit-8
IHC: Immunohistochemistry

## References

[1] Chan BA, Hughes BG (2015). Targeted therapy for non-small cell lung cancer: current standards and the promise of the future. Transl. Lung Cancer Res..

[2] Kim Y (2012). The EGFR T790M mutation in acquired resistance to an irreversible second-generation EGFR inhibitor. Mol. Cancer Ther..

[3] Leung L, Mok TS, Loong H (2012). Combining chemotherapy with epidermal growth factor receptor inhibition in advanced non-small cell lung cancer. Therap. Adv. Med. Oncol..

[4] Tong Y, Huang C, Zhang J (2017). A novel EGFR-TKI inhibitor (cAMP-H3BO3 complex) combined with thermal therapy is a promising strategy to improve lung cancer treatment outcomes. Oncotarget.

[5] Batson S (2017). Tyrosine kinase inhibitor combination therapy in first-line treatment of non-small-cell lung cancer: systematic review and network meta-analysis. OncoTargets Ther..

[6] Jiang T (2017). Addition of bevacizumab for malignant pleural effusion as the manifestation of acquired EGFR-TKI resistance in NSCLC patients. Oncotarget.

[7] Friese-Hamim M, Bladt F, Locatelli G, Stammberger U, Blaukat A (2017). The selective c-Met inhibitor tepotinib can overcome epidermal growth factor receptor inhibitor resistance mediated by aberrant c-Met activation in NSCLC models. Am. J Cancer Res..

[8] Amaravadi RK (2011). Principles and current strategies for targeting autophagy for cancer treatment. Clin. Cancer Res..

[9] Wang Z (2016). Autophagy inhibition facilitates erlotinib cytotoxicity in lung cancer cells through modulation of endoplasmic reticulum stress. Int. J. Oncol..

[10] Yang ZJ, Chee CE, Huang S, Sinicrope F (2011). Autophagy modulation for cancer therapy. Cancer Biol. Ther..

[11] Paglin S (2001). A novel response of cancer cells to radiation involves autophagy and formation of acidic vesicles. Can. Res..

[12] Ertmer A (2007). The anticancer drug imatinib induces cellular autophagy. Leukemia.

[13] Li X, Fan Z (2010). The epidermal growth factor receptor antibody cetuximab induces autophagy in cancer cells by downregulating HIF-1alpha and Bcl-2 and activating the beclin 1/hVps34 complex. Cancer Res..

[14] Han W (2011). EGFR tyrosine kinase inhibitors activate autophagy as a cytoprotective response in human lung cancer cells. PLoS ONE.

[15] Fung C, Chen X, Grandis JR, Duvvuri U (2012). EGFR tyrosine kinase inhibition induces autophagy in cancer cells. Cancer Biol. Ther..

[16] Huang K, Liu D (2016). Targeting non-canonical autophagy overcomes erlotinib resistance in tongue cancer. Tumour Biol..

[17] Liu D, Yang Y, Zhao S (2014). Autophagy facilitates the EGFR-TKI acquired resistance of non-small-cell lung cancer cells. J. Formosan Med. Assoc. Taiwan yi zhi.

[18] Sakuma Y (2013). Enhanced autophagy is required for survival in EGFR-independent EGFR-mutant lung adenocarcinoma cells. Lab. Investig..

[19] So KS (2014). Autophagosome-mediated EGFR down-regulation induced by the CK2 inhibitor enhances the efficacy of EGFR-TKI on EGFR-mutant lung cancer cells with resistance by T790M. PLoS ONE.

[20] Lee TG, Jeong EH, Kim SY, Kim HR, Kim CH (2015). The combination of irreversible EGFR TKIs and SAHA induces apoptosis and autophagy-mediated cell death to overcome acquired resistance in EGFR T790M-mutated lung cancer. Int. J. Cancer.

[21] Lee HR (2016). Poly(ADP-ribosyl)ation is involved in pro-survival autophagy in porcine blastocysts. Mol. Reprod. Dev..

[22] Wang Z, Zhang L, Sagotsky J, Deisboeck TS (2007). Simulating non-small cell lung cancer with a multiscale agent-based model. Theor. Biol. Med. Model..

[23] Munoz-Gamez JA (2009). PARP-1 is involved in autophagy induced by DNA damage. Autophagy.

[24] Barr S (2008). Bypassing cellular EGF receptor dependence through epithelial-to-mesenchymal-like transitions. Clin. Exp. Metas..

[25] Gelmon KA (2011). Olaparib in patients with recurrent high-grade serous or poorly differentiated ovarian carcinoma or triple-negative breast cancer: a phase 2, multicentre, open-label, non-randomised study. Lancet Oncol..

[26] Kleine H (2012). Dynamic subcellular localization of the mono-ADP-ribosyltransferase ARTD10 and interaction with the ubiquitin receptor p62. Cell Commun. Signaling: CCS.

[27] Bokobza SM, Jiang Y, Weber AM, Devery AM, Ryan AJ (2014). Combining AKT inhibition with chloroquine and gefitinib prevents compensatory autophagy and induces cell death in EGFR mutated NSCLC cells. Oncotarget.

[28] Li X, Lu Y, Pan T, Fan Z (2010). Roles of autophagy in cetuximab-mediated cancer therapy against EGFR. Autophagy.

[29] Erguven M (2011). Decreased therapeutic effects of noscapine combined with imatinib mesylate on human glioblastoma in vitro and the effect of midkine. Cancer Cell Int..

[30] Pfaffle HN (2013). EGFR-activating mutations correlate with a Fanconi anemia-like cellular phenotype that includes PARP inhibitor sensitivity. Cancer Res..

[31] Yuan K, Sun Y, Zhou T, McDonald J, Chen Y (2013). PARP-1 regulates resistance of pancreatic cancer to TRAIL therapy. Clin. Cancer Res..

[32] Huehls AM (2011). Poly(ADP-Ribose) polymerase inhibition synergizes with 5-fluorodeoxyuridine but not 5-fluorouracil in ovarian cancer cells. Cancer Res..

[33] Huang Q, Shen HM (2009). To die or to live: the dual role of poly(ADP-ribose) polymerase-1 in autophagy and necrosis under oxidative stress and DNA damage. Autophagy.

[34] Li GH (2015). Ox-Lp(a) transiently induces HUVEC autophagy via an ROS-dependent PAPR-1-LKB1-AMPK-mTOR pathway. Atherosclerosis.

[35] Deeb D (2016). The inhibition of cell proliferation and induction of apoptosis in pancreatic ductal adenocarcinoma cells by verrucarin A, a macrocyclic trichothecene, is associated with the inhibition of Akt/NF-small ka, CyrillicB/mTOR prosurvival signaling. Int. J. Oncol..

[36] Sun Y (2014). Rapamycin-resistant poly (ADP-ribose) polymerase-1 overexpression is a potential therapeutic target in lymphangioleiomyomatosis. Am. J. Respir. Cell Mol. Biol..

[37] Deeb D, Gao X, Liu YB, Gautam SC (2012). Inhibition of cell proliferation and induction of apoptosis by CDDO-Me in pancreatic cancer cells is ROS-dependent. J. Exp. Ther. Oncol..

[38] Deeb D (2010). Growth inhibitory and apoptosis-inducing effects of xanthohumol, a prenylated chalone present in hops, in human prostate cancer cells. Anticancer Res..

[39] Ethier C, Tardif M, Arul L, Poirier GG (2012). PARP-1 modulation of mTOR signaling in response to a DNA alkylating agent. PLoS ONE.

[40] Dean E (2012). Phase I study to assess the safety and tolerability of olaparib in combination with bevacizumab in patients with advanced solid tumours. Br. J. Cancer.

[41] Khan OA (2011). A phase I study of the safety and tolerability of olaparib (AZD2281, KU0059436) and dacarbazine in patients with advanced solid tumours. Br. J. Cancer.

[42] Duran A (2008). The signaling adaptor p62 is an important NF-kappaB mediator in tumorigenesis. Cancer Cell.

[43] Huang J (2018). Adipocyte p62/SQSTM1 suppresses tumorigenesis through opposite regulations of metabolism in adipose tissue and tumor. Cancer Cell.

[44] Mizuno Y (2018). DEPDC5 deficiency contributes to resistance to leucine starvation via p62 accumulation in hepatocellular carcinoma. Sci. Rep..

[45] Wen J (2018). Potentially functional variants of ATG16L2 predict radiation pneumonitis and outcomes in patients with non-small cell lung cancer after definitive radiotherapy. J. Thoracic Oncol..

[46] Bergmann A (2007). Autophagy and cell death: no longer at odds. Cell.

[47] Ceccarelli J (2008). The redox state of the lung cancer microenvironment depends on the levels of thioredoxin expressed by tumor cells and affects tumor progression and response to prooxidants. Int. J. Cancer.

[48] Hagan MP, Yacoub A, Dent P (2007). Radiation-induced PARP activation is enhanced through EGFR-ERK signaling. J. Cell. Biochem..

[49] Hoffman RM (1991). In vitro sensitivity assays in cancer: a review, analysis, and prognosis. J. Clin. Lab. Anal..

[50] Da Broi MG, Jordao AA, Ferriani RA, Navarro PA (2018). Oocyte oxidative DNA damage may be involved in minimal/mild endometriosis-related infertility. Mol. Reprod. Dev..

[51] Wang D (2009). APE1 overexpression is associated with cisplatin resistance in non-small cell lung cancer and targeted inhibition of APE1 enhances the activity of cisplatin in A549 cells. Lung Cancer.

