# Supplementary material for: Role of PARP1-mediated autophagy in EGFR-TKI resistance in non-small cell lung cancer
Source: Sci Rep. 2020 Dec 1;10:20924. doi: 10.1038/s41598-020-77908-z (PMC7708842; doi:10.1038/s41598-020-77908-z)
Supplement: Supplementary file 1 — Supplementary Information 1. [file 41598_2020_77908_MOESM1_ESM.docx]

**Role of PARP1-mediated autophagy in EGFR-TKI resistance in non-small cell lung cancer**

Zhimin Zhang^1*^, Xiaojuan Lian^2*^, Wei Xie^1^, Jin Quan^2^, Maojun Liao^1^, Yan Wu^3^, Zhen-Zhou Yang^3#*^, Ge Wang^1#*^

**Authors' Affiliations**:

^1^ Cancer Center, Daping Hospital, Army Medical University, Chongqing 400042, China;

^2^ Oncology, Jiangjin Strict Central Hospital, Chongqing 402260, China;

^3^ Oncology, Second Hospital Affiliated to Chongqing Medical University, Chongqing 400010, China.

***** These authors contributed equally to this article.

^#^ **Corresponding Author**: Zhenzhou Yang, Department of Oncology, Second Hospital Affiliated to Chongqing Medical University, Chongqing 400010, China, #76 Linjiang Road, Chongqing, China, 400042, China. *E-mail address*: [yangzhenzhou@163.com](mailto:yangzhenzhou@163.com) and Ge Wang, Cancer Center, Daping Hospital, Army Medical University, #10 Changjiang Zhilu, Daping, Yuzhong District, Chongqing 400042, China. *E-mail address*: [wangge](mailto:774022337@qq.com)@126.com.

**Figure. S1**


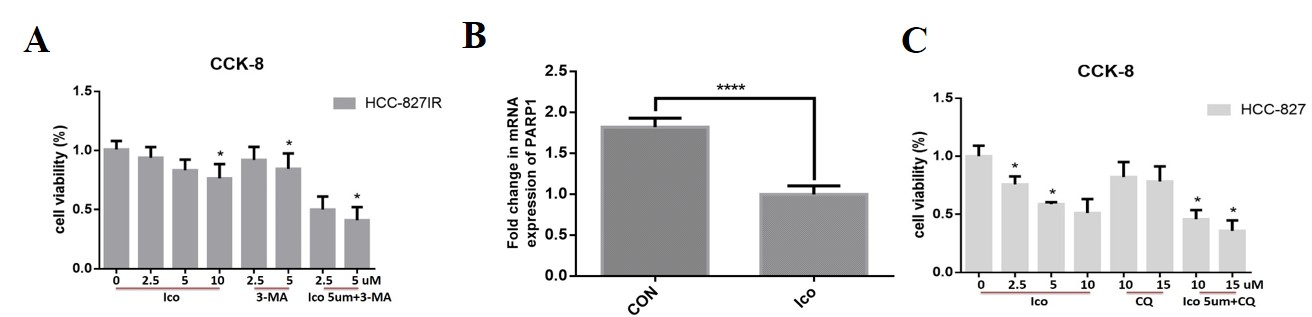


(A) HCC-827IR cells were treated with 0, 2.5, 5, or 10 µM Ico; 2.5 or 5 µM 3-MA (a specific autophagy inhibitors); or a combination of both for 48 h. Cell viability was determined by the CCK-8 assay. (B) The PARP1 mRNA expression level of HCC-827 cells were measured using qRT-PCR in the indicated cells. The indicated cells were transfected with the indicated oligonucleotides or infected with the indicated lentivirus. After 72 h, cells were subjected to qRT-PCR analysis. (C) HCC-827 cells were treated with 0, 2.5, 5, or 10 µM Ico; 10 or 15 µM CQ; or a combination of both for 48 h. Cell viability was determined by the CCK-8 assay. *p < 0.05 vs untreated ****p < 0.0001 vs untreated. The data represent means ± SD of three independent experiments.

**Figure. S2**


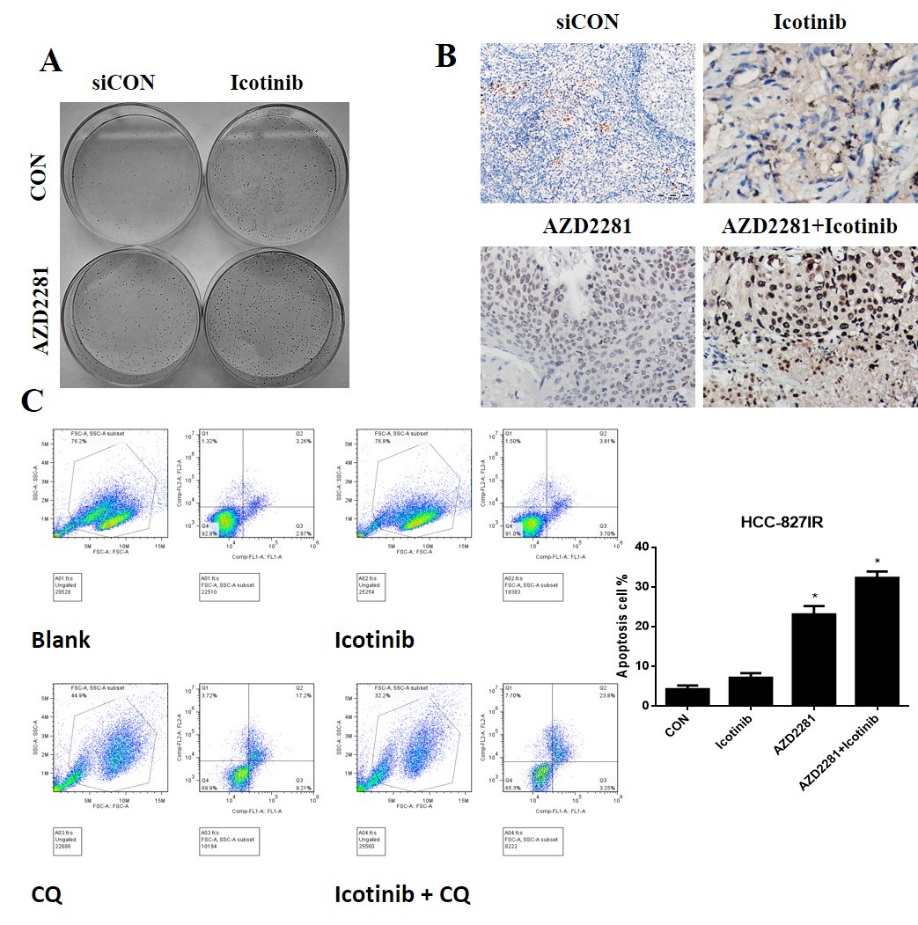


HCC-827IR cells were treated with 5 µM Ico; 4 µM AZD2281; or a combination of both. Colony proliferation assay determined cell proliferation. (B) Nude mouse HCC-827IR xenograft models were divided into four groups (10 mice/group) and treated for 14 days with 50 mg/kg Ico once daily and/or 30 mg/kg AZD2281 once daily by intraperitoneal injection. Tumor tissues isolated from xenografts after treatment with Ico, AZD2281, or Ico + AZD2281 were subjected to immunohistochemical stain of Ki-67 expression. The data shown represent the mean ± standard error, n=10. *P<0.05 versus the vehicle group. (C) HCC-827IR cells were treated with 5 µM Ico; 10 µM CQ; or a combination of both for 24 h. Cell apoptosis were subjected to flow cytometry analysis. The data represent means ± SD of three independent experiments, *p < 0.05 vs untreated.

**Table. Clinical characteristics of NSCLC patients.**

| **Characteristic** | **NO. (%)** |
| --- | --- |
| Age | |
| ≥65 years | 32(27%) |
| ＜65 years | 87(73%) |
| Gender | |
| Male | 65(53%) |
| Female | 54(45%) |
| Histological type | |
| adenocarcinoma | 98(91%) |
| Squamous cell carcinoma | 11(9%) |
| Expression levels of PARP1 | |
| Low (-/+) | 76(64%) |
| High (++/+++) | 43(36%) |
| Expression of p62 | |
| Low (-/+) | 77(65%) |
| High (++/+++) | 42(35%) |
| mutation sites of EGFR | |
| Exon 19 deletion | 56(47%) |
| L858R | 63(53%) |
