# Supplementary material for: Role of PARP1-mediated autophagy in EGFR-TKI resistance in non-small cell lung cancer
Source: Sci Rep. 2020 Dec 1;10:20924. doi: 10.1038/s41598-020-77908-z (PMC7708842; doi:10.1038/s41598-020-77908-z)
Supplement: Supplementary file 2 — Supplementary Information 2. [file 41598_2020_77908_MOESM2_ESM.pdf]

Role of PARP1-mediated autophagy in EGFR-TKI resistance in non-small cell lung cancer

Zhimin Zhang[1*], Xiaojuan Lian[2*], Wei Xie[1], Jin Quan[2], Maojun Liao[1], Yan Wu[3], Zhen-Zhou Yang[3#*], Ge Wang[1#*]

Authors' Affiliations:

[1] Cancer Center, Daping Hospital, Army Medical University, Chongqing 400042, China;

[2] Oncology, Jiangjin Strict Central Hospital, Chongqing 402260, China;

[3] Oncology, Second Hospital Affiliated to Chongqing Medical University, Chongqing 400010, China.

***** These authors contributed equally to this article.

[#] **Corresponding Author**: Zhenzhou Yang, Department of Oncology, Second Hospital Affiliated to Chongqing Medical University, Chongqing 400010, China, #76 Linjiang Road, Chongqing, China, 400042, China. *E-mail address*: yangzhenzhou@163.com and Ge Wang, Cancer Center, Daping Hospital, Army Medical University, #10 Changjiang Zhilu, Daping, Yuzhong District, Chongqing 400042, China. *E-mail address*: wangge@126.com.

Original blots

These below are our original pictures we repeated three times. Because of the films before imaging were cut, we couldn't further provided fuller-length original blots. And, we provided an explanation for the absence of images of adequate length in the figure legends.

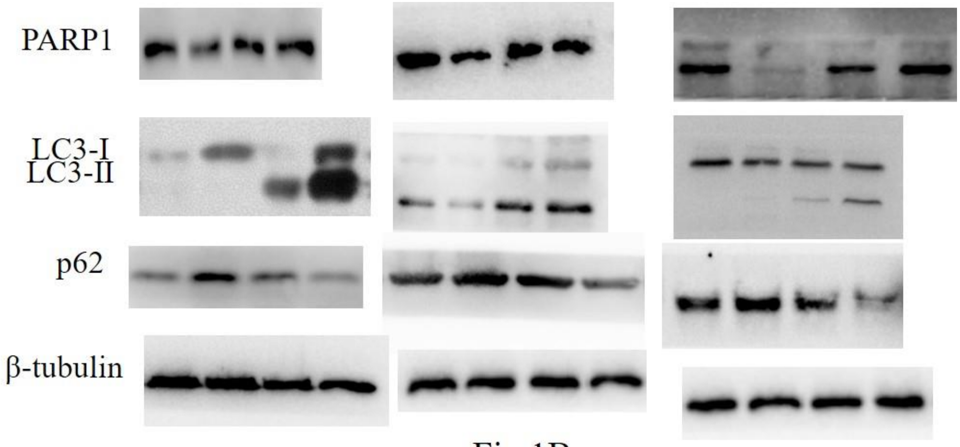

Fig 1B

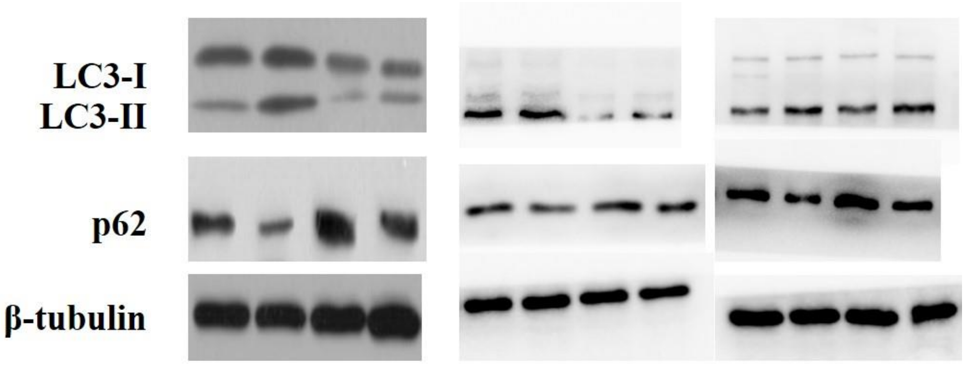

Fig 3C

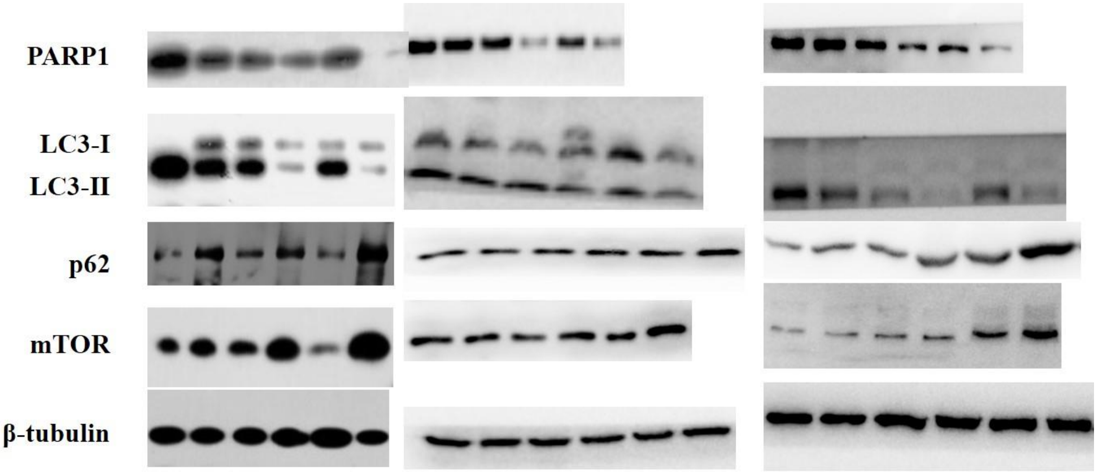

Fig 4A

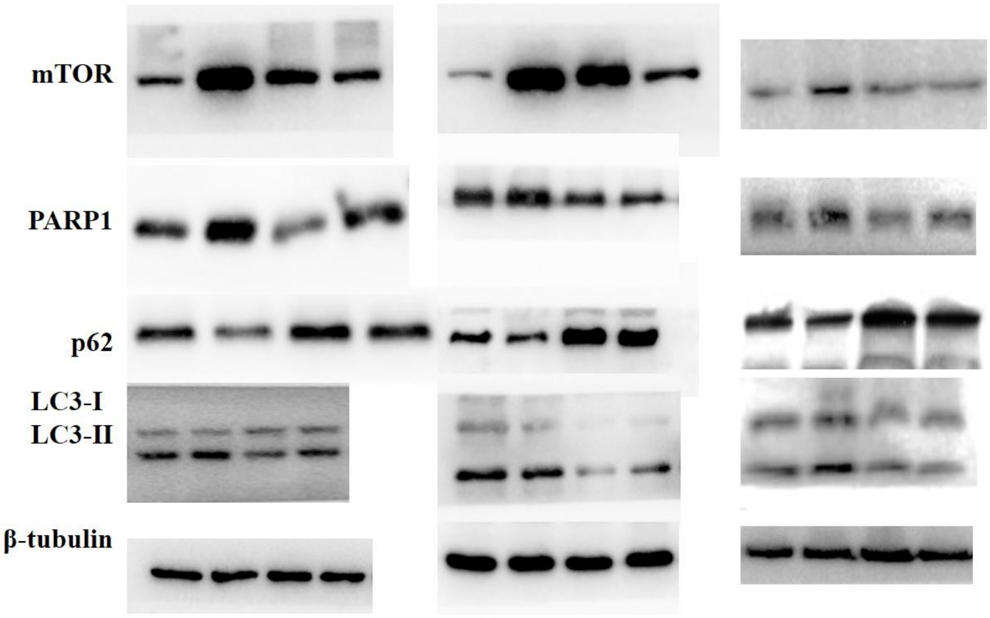

Fig 5C

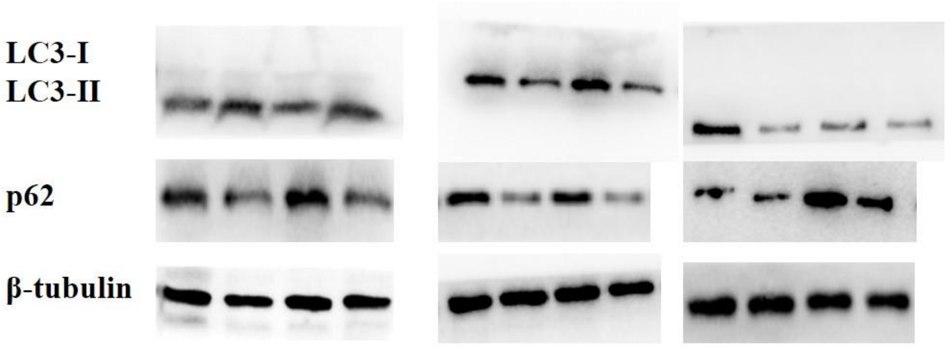

Fig 6B

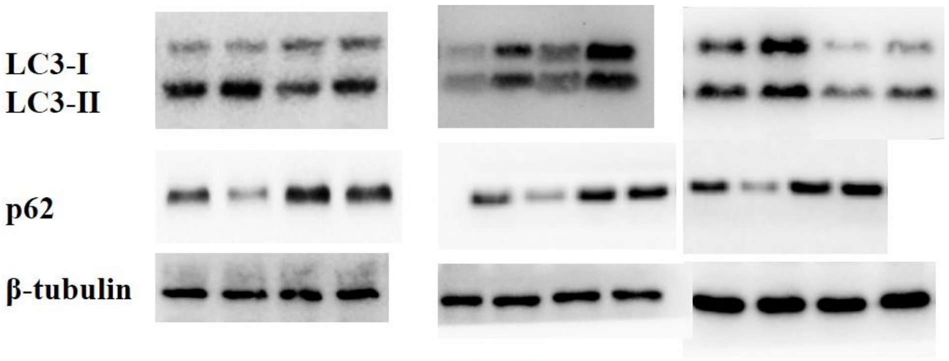

Fig 6D
